# Supplementary figures and images for: Sustainability of an Educational Program on Oral Care/Hygiene Provision by Healthcare Providers to Older Residents in Long-Term Care Institutions: A Follow-Up Study
Source: Geriatrics (Basel). 2024 Jun 20;9(3):84. doi: 10.3390/geriatrics9030084 (PMC11203192; doi:10.3390/geriatrics9030084)

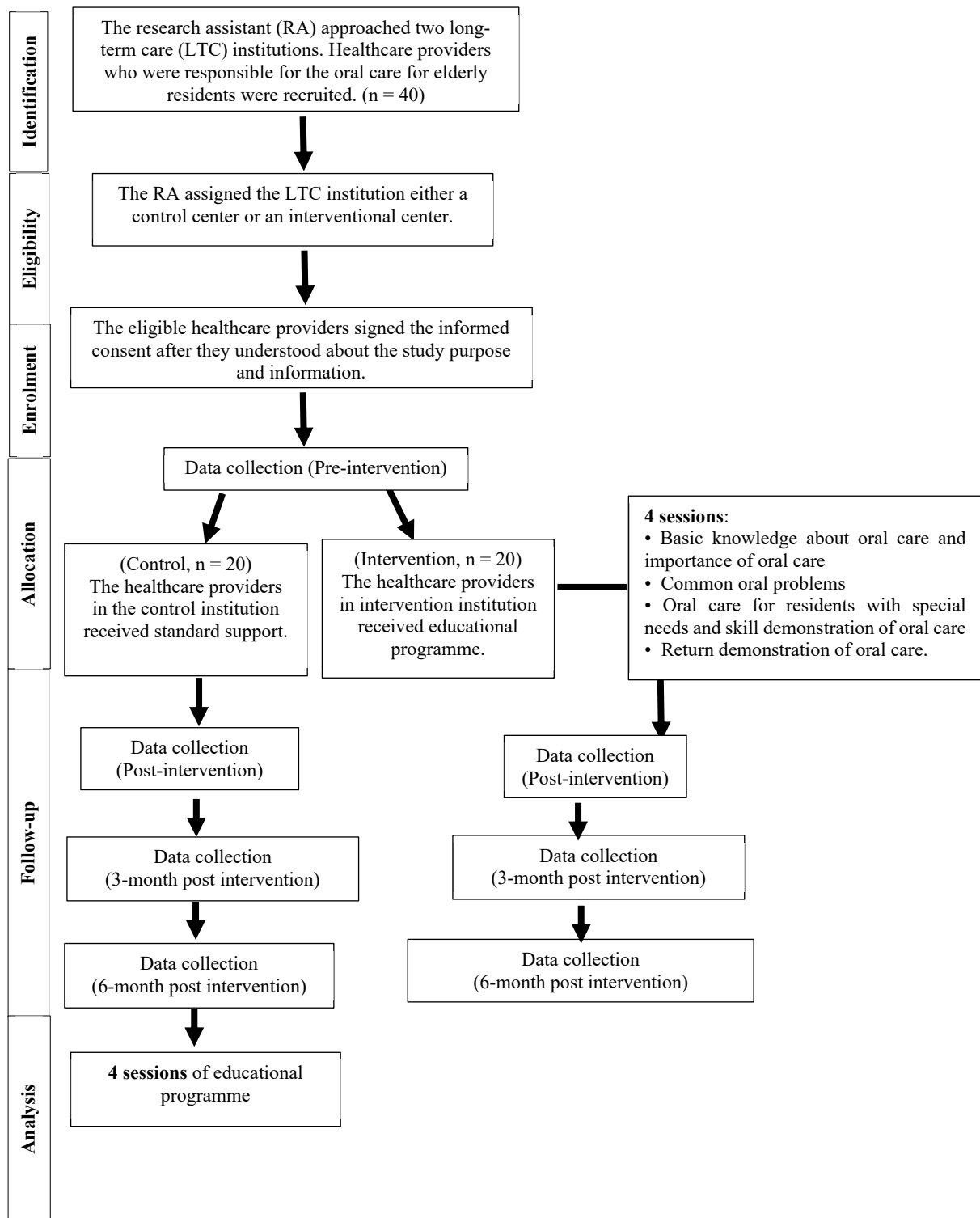

**Figure S1.** The flow of the study.

Supplement: Supplementary file 1 [file geriatrics-09-00084-s001.zip › geriatrics-2957672-supplementary.pdf]
